# A commentary on ‘Why public health cannot be led by doctors only’

**DOI:** 10.1097/JS9.0000000000000465

**Published:** 2023-05-15

**Authors:** Si-Un Frank Chiu, Kuo-Chuan Hung, Chong-Chi Chiu

**Affiliations:** a Department of Computer Science; b Department of Economics, Brown University, Providence, Rhode Island, USA; c Department of Anesthesiology, Chi Mei Medical Center; d Department of Hospital and Health Care Administration, College of Recreation and Health Management, Chia Nan University of Pharmacy and Science, Tainan; e Department of General Surgery; f Department of Medical Education and Research, E-Da Cancer Hospital; g School of Medicine, College of Medicine, I-Shou University, Kaohsiung, Taiwan

*Dear Editor,*

The issue of leadership in public health has been a point of debate for a long time. Yasin *et al*.^1^ have raised many reasons to support the idea that public health cannot be led by doctors alone. However, many specialists still believe that doctors should primarily hold this position as they have the necessary medical expertise and knowledge to identify and tackle societal health problems. Public health is a crucial aspect of society that requires the guidance and direction of these experienced medical professionals to develop policies and strategies to maintain the quality of life of individuals and communities. Doctors have the necessary skills and training to analyze society’s health problems and formulate strategies to address them. They also have the experience and knowledge to collaborate with domestic and international stakeholders to solve public health problems^2^.

Public health is a complex field that requires a multidisciplinary approach^3^. While doctors may have expertise in only some of the core areas of public health, they have a solid understanding of medical issues and are trained to think critically and solve complex problems. Therefore, many doctors are believed to contribute value to public health leadership.

Leadership skills are not necessarily a weak point in doctors. On the contrary, they are essential to a medical professional’s training and are taught throughout the medical curriculum. Medical students in many countries already receive training in leadership skills^4^. Thus, most doctors can lead teams, make decisions, and manage resources, all of which are crucial skills for effective leadership in public health. In other words, revising medical curriculums to teach leadership skills is unnecessary. Although some doctors may need more leadership skills, it is not fair to generalize and assume that all doctors are deficient in this area.

There may be different fields of public health, such as medical, biological, behavioral, environmental, and social medicine, mentioned in the text^1^. Most doctors have the expertise and knowledge to navigate and integrate these fields effectively. Besides, the argument that medical leaders only focus on secondary or tertiary prevention and neglect primary prevention is unfounded^5^. Doctors are trained to diagnose and treat medical problems, but they also understand the vital significance of primary prevention of diseases, and they could use this knowledge to develop policies that prevent the occurrence of diseases in communities. Doctors could also work collaboratively with other professionals in public health to develop strategies for preventing diseases and promoting public health, as they usually do to build a treatment plan for a patient.

Furthermore, it is not accurate to say that public health has suffered because of the dominance of doctors. Doctors have played a crucial role in public health leadership throughout history, and they continue to do so today in most countries. The idea that public health has been divided and dispersed among the power rule by one dominant discipline is flawed. On the other hand, public health is a collaborative effort that requires the contributions of professionals from various fields.

Overall, it is essential to recognize that doctors and other experienced medical professionals are indispensable in public health leadership. While having a multidisciplinary approach to public health is essential, doctors have valuable knowledge and expertise that can contribute to public health leadership.

Healthcare professionals apply behavioral health principles, community-based practise, and environmental health to guide policies and procedures. The practise of public health should focus on evidence-based interventions that can improve health, prolong life, and prevent various illnesses. Thus, public health leaders should be chosen based on merit and expertise rather than on preconceived notions about who can or cannot be a good leader.

## Ethical approval

This is only a commentary, not research involving patients. No ethical approval is required.

## Consent

This is only a commentary, not research involving patients. No patient consent is required.

## Sources of funding

This is a commentary. We have no funding for our commentary.

## Conflicts of interest disclosure

There are no conflicts of interest in our commentary.

## Research registration unique identifying number (UIN)

This is a commentary on a study published in the ‘International Journal of Surgery’. No UIN is required.

## Author contribution

S.-U.F.C.: conceptualization and writing original draft; K.-C.H.: validation; C.-C.C.: conceptualization, supervision, submission, and correspondence.

## Guarantor

Professor Chong-Chi Chiu.

## Peer review

This paper was not invited.

## Data availability statement

This is only a commentary on a study published in the ‘International Journal of Surgery’. There are no research data in our commentary.
